# Expansion and Harvesting of hMSC-TERT

**DOI:** 10.2174/1874120700701010038

**Published:** 2007-09-07

**Authors:** Christian Weber, Sebastian Pohl, Ralf Pörtner, Christine Wallrapp, Moustapha Kassem, Peter Geigle, Peter Czermak

**Affiliations:** 1 Institute of Biopharmaceutical Technology, University of Applied Sciences Giessen-Friedberg, Giessen-Germany; 2 Institute of Bioprocess and Biosystems Engineering, University of Technology, Hamburg-Germany; 3 CellMed AG, Alzenau-Germany; 4 Department of Endocrinology and Metabolism, University Hospital of Odense, Odense-Denmark5; 5 Department of Chemical Engineering, Kansas State University, Manhattan KS-USA

**Keywords:** Harvest, hMSC-TERT, mesenchymal stem cells, microcarrier, spinner flask.

## Abstract

The expansion of human mesenchymal stem cells as suspension culture by means of spinner flasks and microcarriers, compared to the cultivation in tissue culture flasks, offers the advantage of reducing the requirements of large incubator capacities as well as reducing the handling effort during cultivation and harvesting. Nonporous microcarriers are preferable when the cells need to be kept in viable condition for further applications like tissue engineering or cell therapy. In this study, the qualification of Biosilon, Cytodex 1, Cytodex 3, RapidCell and P102-L for expansion of hMSC-TERT with an associated harvesting process using either trypsin, accutase, collagenase or a trypsin-accutase mixture was investigated. A subsequent adipogenic differentiation of harvested hMSC-TERT was performed in order to observe possible negative effects on their (adipogenic) differentiation potential as a result of the cultivation and harvesting method. The cultivated cells showed an average growth rate of 0.52 d^-1^. The cells cultivated on Biosilon, RapidCell and P102-L were harvested succesfully achieving high cell yield and vitalities near 100%. This was not the case for cells on Cytodex 1 and Cytodex 3. The trypsin-accutase mix was most effective. After spinner expansion and harvesting the cells were successfully differentiated to adipocytes.

## INTRODUCTION

Because of their multilineage potential [1], human mesenchymal stem cells are qualified for many applications such as tissue engineering and cell therapy [2-6]. Normally, the cells are expanded before their use. The expansion of hMSC in tissue culture flasks is a widely used method but it is limited because of the restricted growth surface. This results in the need for large incubator capacities and extensive benchtop work to achieve high numbers of cells. Suspension cultures on the basis of microcarriers are an alternative to culture flasks because of their high growth surface per volume and therefore a greater achievable spatial yield [7]. Many investigations on microcarriers mainly with respect to their yields of secreted biomolecules or viruses as product of the cultivation process have been published [8-11]. If the aim of the cultivation process is a high number of viable cells, for instance for cell therapy, special needs such as a good harvesting behavior, high vitality after harvesting, and a conserved differentiation potential after cultivation and harvesting arise. It is obvious, that macroporous microcarriers are not suitable because of the hindered separation of the enzymatically detached cells inside of the carrier. This disadvantage can be avoided by using nonporous microcarriers such as Cytodex, Biosilon or Rapidcell. Beside the geometrical properties of microcarriers, surface properties like charge and roughness have a significant influence on detachment and thus on the cell yield and vitality after the enzymatic treatment.

The aim of this work was the investigation of the suitability of various spherical nonporous microcarriers (Table **1**) for cultivation of hMSC in spinner flasks with a subsequent harvesting procedure using trypsin, accutase and collagenase. The yield and the vitality of the cells after harvesting are of particular interest. One carrier with good growing and harvesting features was selected for an investigation of the adipogenic differentiation potential after cultivation in spinner flasks and harvesting using the various enzymes. Experiments were additionally performed with cells grown in 6-well cell culture plates. These are very simple devices for cultivation of adherent cells as an alternative to tissue culture flasks. Cell cultures usually show good growth behavior in these culture dishes, which are commonly made from polystyrene. Because of their low surface to volume ratio, and therefore significant physical space requirement cell culture plates are only useful for laboratory scale work or to prepare inoculi. Therefore, the growth and harvesting characteristics in 6-well cell culture plates were used as reference for techniques using microcarriers and spinner flasks.

Human mesenchymal stem cells, which were transduced by a retroviral vector, containing the catalytic subunit of human telomerase (hTERT), were used in this work. The hTERT-subunit counteracts the shortening of the telomers after each cell cycle and leads thereby to an increase of the achievable population doublings [12]. The hMSC-TERT cell line was choosen as the model cell line because only small or no changes in their attributes during an increasing number of passages were expected. This allowed a comparison of experimental results with cells of different passage numbers.

## MATERIALS AND METHODS

All chemicals were obtained from Sigma-Aldrich (Deisenhofen, Germany) unless otherwise indicated.

The hMSC-TERT cell line was provided by Prof. Dr. Moustapha Kassem (University Hospital of Odense, Denmark). The passage numbers throughout the experiments was between 70 and 75.

### Media

The applied culture medium was EMEM (Biochrom AG, Berlin, Germany), supplemented with 10% BGS (Bovine Growth Serum, Thermo Fisher Scientific, Schwerte, Germany), 100 U/ml penicillin and 100 µg/ml streptomycin. It was used during cultivations without adipogenic differentiation.

Induction medium was used to induce the adipogenic differentiation. The induction medium consisted of DMEM-HG (Biowest, Nuaille, France), that was supplemented with 10 % BGS, 100 U/ml penicillin and 100 µg/ml streptomycin, 1 μM dexamethason, 0.2 mM indomethacin, 0.01 mg/ml insulin and 0.5 mM 3-isobutyl-1-methyl-xanthin [13-15].

DMEM-HG supplemented with 10% BGS, 10 mg/l insulin, 100 U/ml penicillin and 100 µg/ml streptomycin was used as maintenance medium between the induction phases of adipogenic differentiation.

### Crystal Violet Assay

The cell density in the microcarrier suspensions has to be determined for calculation of the yield after harvesting. Therefore a crystal violet assay was used to stain the nuclei after cell lysis.

1.5 ml microcarrier suspension was transferred into a 1.5 ml reaction tube. 1 ml supernatant was discarded and replaced by 1 ml crystal violet solution after sedimentation of the microcarriers. The tubes were incubated for 24 hours and centrifuged for 2 minutes at 200 Xg. The nuclei were counted using a Neubauer counting chamber.

The crystal violet solution was prepared by dissolving 21 g citric acid and 1 g crystal violet in 1000 ml deionized water. The solution was filtered (0.22 µm) for clarification.

### Oil Red O Staining

Oil red O is a dye suitable for staining lipid droplets and thus may be used to verify the adipogenic differentiation status of the hMSC-TERT.

The medium in the wells of the cell culture plates was discarded and the cells were rinsed three times with PBS. Afterwards the cells were fixed by adding of 1 ml methanal and incubation for one hour. After the removal of the methanal, followed by three washing steps with PBS, the cells were incubated again with 400 µl oil red O solution per well for one hour in the dark. Images were taken by means of a phase contrast microscope (DMIL, Leica AG, Wetzlar, Germany).

The oil red O stock solution was prepared by dissolving 0.7 g oil red O in 200 ml isopropanol and subsequent filtration (0.22µm). The working solution was made by adding 4 ml deionized water to 6 ml oil red O stock solution [16].

### Microcarriers

The non-porous spherical microcarriers are listed in Table **1**. Light microscope images of cell covered microcarriers are shown in Fig. (**1**).

### Enzymes

The utility of the proteases trypsin, accutase and collagenase for detaching of the adherent hMSC-TERT was examined in this study. Porcine trypsin (2.5 mg/ml) and accutase were obtained as ready to use EDTA-containing solution. Trypsin and accutase solutions were both used separately and as a 1:1 (w/w) mixture.

Collagenase (2.5 FALGPA units/mg) was dissolved at 2.5 mg/ml in PBS immediately before use.

### Cultivation in 6-Well Cell Culture Plates

hMSC-TERT were seeded at concentrations of 2500, 5000 and 10,000 cells/cm^2^ into two 6-well cell culture plates. 3 ml culture medium was added to every well and the plates were cultured for 6 days in a humidified incubator (37°C, 5% CO_2_). Every 1-2 days two wells of each cell density were harvested by adding 1 ml trypsin-EDTA solution and incubating them for 5 minutes. Prior to this, the cultivation medium was discarded and the cells were washed with PBS. The cell number was determined with a Neubauer counting chamber. The data were used for the preparation of growth curves. Growth rates were calculated from the exponential phases of the growth curves by curve fitting.

### Harvesting from 6-Well Cell Culture Plates

The investigation of the detachment efficiency of trypsin and accutase was performed in 6-well cell culture plates with subconfluent cells (Fig. **13a**). After medium removal and two washing steps using PBS, 1 ml enzyme solution (trypsin or accutase) was added per well. The culture plates were incubated (37°C) for up to 6 minutes. The reactions were stopped at 30, 60, 90, 120, 240 and 360 seconds by adding 1 ml of serum containing culture medium. Detached cells were counted with a Neubauer counting chamber. The vitality was determined by the trypan blue exclusion method. Each enzyme and reaction time combination was examined in duplicate (two wells each). 15 minutes incubation enabled the determination of the entire cell density and therefore a calculation of the yield.

The statistical means and standard deviations were calculated by Gauss’ law of error propagation.

### Cultivation in Spinner Flasks

hMSC-TERT were cultured in spinner flasks with 250 ml culture medium. Microcarriers with an equivalent growth surface of 8100 cm^2^ were added to the flasks. Seeding densities were about 5000-7000 cells/cm^2^. The spinner flasks were placed on a magnetic stirrer in a humidified (37°C, 5% CO_2_) incubator. To enable the attachment of the cells to the carrier, the agitation (30 rpm) was started with a delay of 4 hours. Medium was changed every 2-3 days. For each microcarrier one cultivation was performed. Daily samples (duplicates) were taken for preparation of growth curves. Cell numbers were determined using the crystal violet assay. The growth rates were calculated from the exponential phase of the growth curves by curve fitting.

### Harvesting from Microcarriers

Good vitality and separation of the cells from the carrier is needed in many applications in addition to a high cell yield . Hence, the qualification of trypsin, accutase, trypsin-accutase mixture and collagenase, combined with a sieving procedure, was investigated in regard to the yield and vitality of the cells. 5 ml microcarrier suspension (corresponding to a growth surface of 40.5 cm^2^) for each carrier-enzyme combination was removed from the spinner flask. The carriers were separated from the ambient medium using a 100µm sieve (Cell Strainer, Becton Dickinson GmbH, Heidelberg, Germany), rinsed with PBS twice and then, still located in the sieve, incubated (37°C) in 4.5 ml enzyme solution for 10 minutes (trypsin, accutase, and trypsin-accutase mixture) or 40 minutes (collagenase). The reaction was stopped by adding 9 ml culture medium. The microcarriers were then rinsed twice with the mixture of culture medium and enzyme solution. The number of detached and separated cells as well as the vitality were determined with a Neubauer counting chamber and the trypan blue exclusion method. Three samples of 5 ml of each microcarrier suspension were incubated in crystal violet solution to get the entire cell number and with it the yield. All experiments were performed in duplicate. The statistical means and standard deviations were calculated using Gauss’ law of error propagation.

### Vitality of the hMSC - Trypan Blue Staining

The trypan blue exclusion method was applied for the quantitative evaluation of the vitality of hMSC-TERT after the harvesting procedures. Trypan blue is able to enter dead cells and stain them blue.

100 µl cell suspension was mixed with 100 µl trypan blue solution (Biowest). After incubation at ambient conditions for about 5 minutes, the numbers of vital and dead cells were determined with a Neubauer counting chamber.

### Adipogenic Differentiation

Cells, harvested with accutase, trypsin and the trypsin-accutase mixture by incubating for 10 minutes in the enzyme solution and separating using a 100µm sieve, were centrifuged at 200 x g for 5 min and resuspended in culture medium. After this, 5000 cells from each probe were seeded into two wells of a 6-well cell culture plate containing 3 ml of culture medium. They were left in incubation until confluence was reached. The medium was changed every 2-3 days. Then, induction medium was applied to one well of each set for 3 days, followed by 4 days cultivation with maintenance medium. This cycle was repeated about 2.5 times (16 days). The cells in the second wells were cultured with maintanance medium only as negative control. Because of the higher sodium bicarbonate content in DMEM, the CO_2_ concentration in the incubator was increased to 10% to maintain a physiological pH. Additional reference cultivations with cells grown in tissue culture flasks and harvested using the accutase-trypsin mixture were performed in the same way (above) to exclude any possible effect of the cultivation in the spinner flasks on the adipogenic differentiation potential.

## RESULTS AND DISCUSSION

### Growth Kinetics

**Fig. (2):**
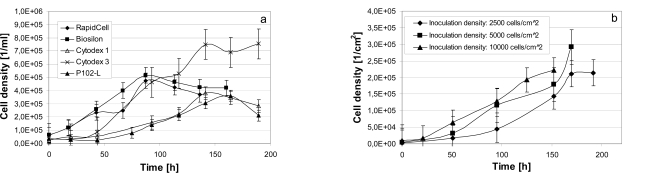
Growth curves of hMSC-TERT, cultured in spinner flasks on diverse nonporous microcarriers (**a**) and in 6-well cell culture plates at inoculation densities of 2500, 5000 and 10,000 cells/cm^2^ (**b**).

Lag-phases of at least 44 hours were observed with Cytodex 1 and P102-L. Cytodex 3 showed a lag-phase of at least 20 hours. RapidCell and Biosilon showed the lowest lag-phase of less than 18 hours (Fig. **2a**). The lag-phases in 6-well cell culture plates were less than 21 hours (Fig. **2b**). We suspect, that the negatively charged growth surfaces reduce the lag-phase of hMSC-TERT.

The cell densites obtained in the spinner flasks were about 450,000 cells/ml on average and could perhaps be increased by enhancing the microcarrier concentration. Related to the growth surface of 8.1 cm^2^ per milliliter microcarrier suspension, an averaged cell density of about 56,000 cells/cm^2^ was reached, which was clearly lower than the achieved cell density in 6-well cell culture plates of up to 292,000 cells/cm^2^ (after 9 days of culture). A possible explanation is that the hMSC-TERT did not stop growing at confluence but rather grew to some degree into multilayers (Fig. **13b**). Such multilayer growth could not be detected in spinner cultures but only between agglomerated microcarriers. This could be due to a shear stress induced detachment by disruption of a fragile bond to the growth surface of post-confluent multilayered cells in agitated spinner flasks. Common hMSC do not grow in multilayers because proliferation stops by contact inhibition at confluence.

**Fig. (3):**
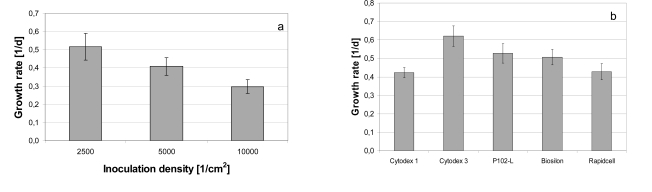
Growth rates of hMSC-TERT (**a**) cultured in 6-well cell culture flasks at different seeding densities and (**b**) on various microcarriers in spinner flasks.

The hMSC-TERT, cultured in 6-well cell culture plates, showed a growth rate of 0.30 ± 0.04 d^-1^ (inoculation density 10,000 cells/cm^2^), 0.40 ± 0.03 d^-1^ (inoculation density 5000 cells/cm^2^) and 0.52 ± 0.07 d^-1 ^at an inoculation density of 2500 cells/cm^2^ (Fig. **3a**).

The growth rate in 6-well cell culture plates increased with decreasing cell density. Similar results, but with non modified hMSC, were published by Weber *et al*. and Both *et al*. [17, 18]. The cell-cell interactions during the early stages of cultivation, which were decreased at lower seeding densities, could have been responsible for this effect. This points towards a possible enhancement of growth rate in spinner cultures by reduced inoculation density. However, very low seeding density could lead to negative effects such as an extended lag-phase [17, 18].

Compared to the growth rates in 6-well cell culture plates at inoculation densities of 5000 and 10,000 cells/cm^2^, the cultivations on microcarriers in spinner flasks with seeding densities between 5000 and 8000 cell/cm^2^ resulted in higher growth rates between 0.42 ± 0.06 d^-1^ and 0.62 ± 0.06 d^-1^ (Fig. **3b**).

Conget and Minguell found a doubling time for hMSC of 33 hours, which corresponds to a growth rate of 0.5 d^-1^ and is similar to the average growth rate reached in this study [20].

The increased growth rates in spinner flasks may be due to lower oxygen and nutrition gradients caused by agitation, in addition to the different surface properties of the microcarriers [21]. This is supported by the growth rate in 6-well cell culture plates at seeding densities of 5000 and 10,000 cell/cm^2^ which is lower than on Biosilon despite the same carrier material (polystyrene), surface charge, and range of seeding density.

### Cell Harvest

**Fig. (4):**
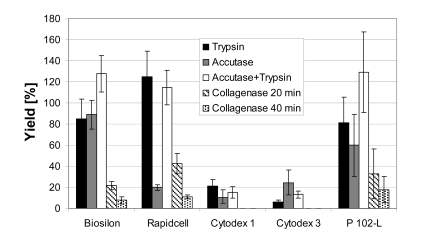
Cell yield after harvesting of hMSC-TERT, cultered in spinner flasks on various nonporous microcarriers with different enzymes.

Cell harvesting with a high yield is possible using all microcarriers except Cytodex 1 and Cytodex 3 (Fig. **4**). Yields of about 100% could be reached using trypsin (Rapidcell) or the trypsin-accutase mix (Biosilon, Rapidcell, P102-L). 100% equals a complete detachment and separation of the cells. For some microcarrier-enzyme combinations, the yield exceeds 100%, which is likely due to experimental uncertainty and error propagation due to the hindered sampling of the rapidly settling microcarriers and the application of two different methods of cell number count (crystal violet assay and counting of viable respectively dead, trypan blue stained cells). However, the excursions above 100% are within a single (1σ) or at least two (2σ) standard deviations.

The enzymes trypsin and accutase were similar regarding their qualification for cell harvesting. The effectiveness of the enzymes appeared to depended on the microcarrier-enzyme combination. However, it appears clear that the combination of trypsin and accutase was, at least for treatment of RapidCell, Biosilon, and P102-L, more effective than using the enzymes separately.

The low yield using collagenase might be increased by higher enzyme activities (concentrations) and longer reaction times. Especially the cell harvest from the collagen coated Cytodex 3 could perhaps be improved by digestion of the collagen coating. With collagenase concentration and reaction times applied here no yield was obtained by application to Cytodex 1 and Cytodex 3.

Fig. (**5**). shows the yield of hMSC-TERT cultured in 6-well cell culture plates and harvested using trypsin and accutase solution at the same volume-surface ratio after different reaction times. 100% yield was reached after 120 seconds by accutase whereas with trypsin a complete detachment of cells was achieved after 360 seconds. The incubation of Biosilon for 10 minutes with accutase or trypsin led to a yield of only 85-89%, despite similar surface characteristics of Biosilon and 6-well cell culture plates (polystyrene, negatively charged). However, the combination of both trypsin and accutase overcomes this disadvantage. Furthermore, longer incubation with trypsin or accutase may increase the cell yield, but this may cause degradation of adhesion and receptor proteins. This could be unfavorable in subsequent applications of the cells.

The Figs. (**6-10**) are similar to the results in Fig. (**4**). The incubation of Cytodex 1 and Cytodex 3 with the enzyme solutions for 10 minutes did not lead to a significant detachment of the cells, especially with collagenase.

The low yields using collagenase in all cases suggest a low detaching potential, but it can be seen in Fig. (**6**, **7, 10**), that the cells form aggregates which did not pass through the cell strainer and hence the apparent cell yield was reduced. This supports the assumption that a higher collagenase activity as well as an increased incubation time and larger mesh aperture may counteract this problem. The use of collagenase for the detachment of cells offers the advantage of reducing membrane protein damage due to the enzymes specificity for collagen.

**Fig. (11):**
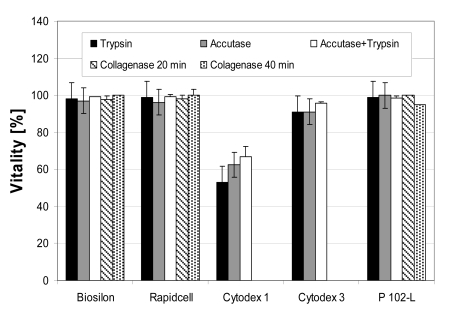
Vitalities of cultured hMSC-TERT on nonporous microcarriers immediately after the harvesting procedure. The vitality was determined by the trypan blue exclusion method.

**Fig. (12):**
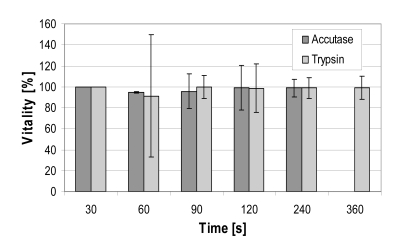
Vitalities of cultured hMSC-TERT in 6-well cell culture plates after incubation with trypsin an accutase for up to 6 minutes. Vitality was determined using the trypan blue exclusion method.

High vitalities of about 100% were obtained in most cases with exception of the harvest from Cytodex 1 and Cytodex 3, which showed a reduced vitality (Fig. **11** and **12**). This is explained by the low cell yield and the thereby larger percentage of easily detached morbid cells.

The vitality was determined immediately after the harvesting procedure by the trypan blue exclusion method, which detects the fraction of dead cells related to the number of totally detached and separated cells. This method does not predict the viability of the cells in a new culture. It is possible, that some cells will die later during a subsequent cultivation process for example due to damaged membrane proteins. The vitalities showed serious damage, which could lead to a rapid cell death.

**Fig. (13):**
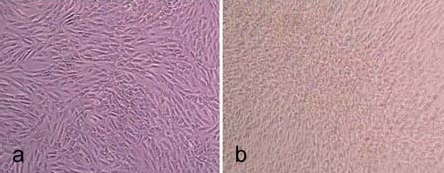
hMSC-TERT on the 4th day of culture (**a**) directly prior to the enzyme treatment and on the 8th day (**b**) of culture.

**Fig. (14):**
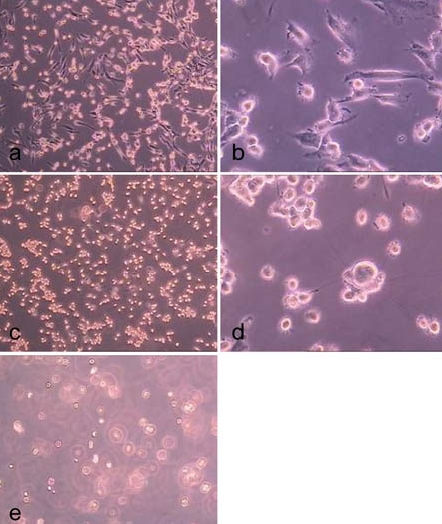
Treatment of hMSC-TERT, grown in 6-well cell culture plates, with accutase for 0.5 min (**a**, **b**), 1.0 min (**c**, **d**) and 2.0 min (**d** ,**e**). (light microscope images, magnification factor 100 and 200).

**Fig. (15):**
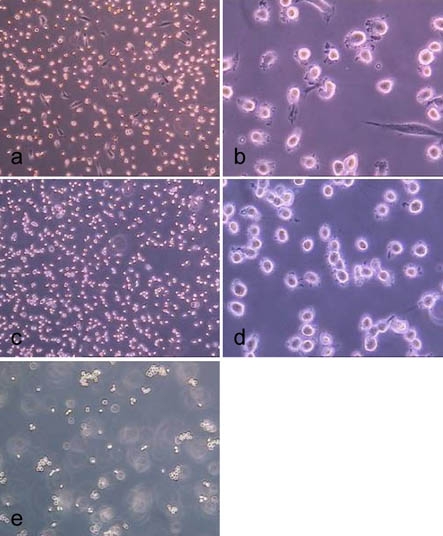
Treatment of hMSC-TERT, grown in 6-well cell culture plates, with trypsin for 0.5 min (**a**, **b**), 1.0 min (**c**, **d**) and 2.0 min (**d**, **e**). (light microscope images, magnification factor 100 and 200).

Figs. (**13** to **15**) show the cells in 6-well cell cultures plates directly before (Fig. **13**) and during (Fig. **14** and **15**) the treatment with trypsin and accutase. Fig. (**14d** and **15d**) demonstrate that the cells become spherical and suggest to be detached but are still fixed to the growth surface by means of barely visible membrane protuberances. This means, that the yield by harvesting cells from microcarriers could be increased without a significant increase of enzyme reaction time simply by inducing some shear stress, which disrupts these protuberances. How far this treatment might reduce the viabilty of the cells is a target of further investigation.

### Adipogenic Differentiation

Biosilon was chosen for the investigation of the adipogenic differentiation potential of hMSC-TERT, grown on this microcarrier and harvested using trypsin, accutase or the trypsin-accutase mix. The goal was to exclude possible negative effects on the adipogenic differentiation potential due to the cultivation in spinner flasks and due to the harvesting procedure.

**Fig. (16):**
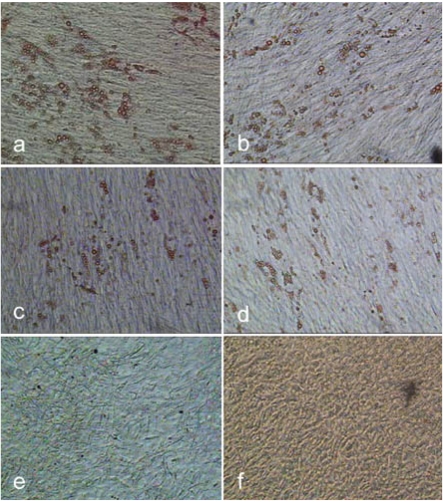
Oil red O staining of hMSC-TERT after adipogenic cultivation for 16 days. The cells were cultured in spinner flasks and harvested by means of accutase (**a**), trypsin (**b**) and a 1:1 (w/w) trypsin accutase mix (**c**) before adipogenic cultivation in 6-well cell culture plates. hMSC-TERT cultured in tissue culture flasks and harvested with the trypsin-accutase mix shown as reference (**d**). Negative controls without adipogenic stimulation were performed with cells, which were grown in a spinner (**e**) and tissue culture flask (**f**) and harvested using the trypsin-accutase mixture.

The adipogenic cultured cells, previosly expanded in spinner flasks, showed the formation of lipid droplets, which indicates the differentiation into adipocytes (Fig. **16a**-**c**). There is no negative effect visible on the adipogenic differentiation compared to the cells, which were previously expanded in tissue culture flasks. Thus, the cultivation in spinner flasks may be considered as a practical option for the expansion of the hMSC-TERT with a subsequent and at least adipogenic differentiation. The different numbers of lipid droplets containing cells in Fig. (**16a-d**) suggest an influence of the type of harvesting enzyme on the adipogenic differentiation potential. It should be considered, that the adipogenic assay is only a qualitative assessment. Furthermore the relatively low yield of differentiated cells suggests a low or no adipogenic differentiation of a large number of the cells. This may be due to a reduced adipogenic cultivation period of 16 instead of 21 days [14], because of an incipient detachment of the cells and hence a premature interruption of the differentiation process. The cells received 5 days less for differentiation and formation of lipid droplets. Increased cultivation time might have resulted in an enhanced formation of lipid droplets. Another influence on the differentiation of animal cells is the serum lot and/or previous treatment of the serum [22-24]. It would therefore be useful to test the serum lot before application [23]. The type of medium may also affect the differentiation of hMSC too [17].

The investigation of the adipogenic differentiation was used as an example. The potential to differentiate into other mesenchymal lineages after the cultivation and harvesting should be investigated in further studies to exclude a possible reduction of the differentiation potential.

## CONCLUSION

The aim of this study was the investigation of the suitability of nonporous microcarriers for the expansion of hMSC-TERT. The usefulness of trypsin, accutase, collagenase and a 1:1 (w/w) trypsin-accutase mixture was also evaluated with the target of high cell yield and vitality after harvesting. A subsequent adipogenic cultivation of the cells, which had been expanded in spinner flasks on Biosilon and in tissue culture flasks and harvested with the different enzymes or enzyme mixtures was performed to exclude negative effects of the cultivation and harvesting procedure on the (adipogenic) differentiation potential.

The cells cultured on microcarriers in spinner flasks, showed an average growth rate of 0.52 d^-1^. The cells grown in 6-well cell culture plates showed growth rates between 0.3 and 0.52 d^-1^. Decreased seeding density resulted in an increased growth rate. The cells were harvested succesfully with yields and vitalities up to 100% from Biosilon, Rapid-Cell and P102-L but not from Cytodex 1 and Cytodex 3. The trypsin-accutase mixture was most effective. The cells could be differentiated to adipocytes after spinner expansion and harvesting procedure. An optimization of the differentiation protocol to increase the yield of differentiated hMSC-TERT should be carried out.

## Figures and Tables

**Fig. (1):**
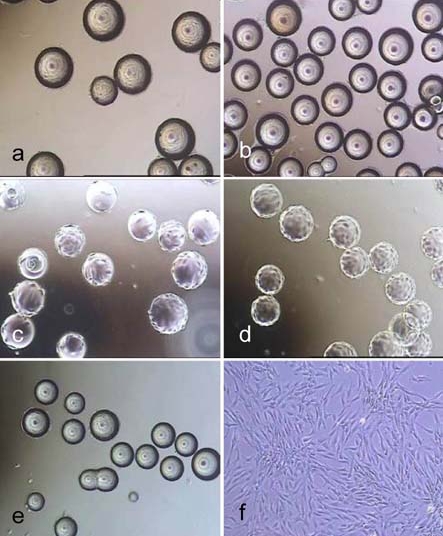
Light microscope images of hMSC-TERT, cultured in spinner flasks (a-e) on Biosiln (a), RapidCell (b), Cytodex 1 (c), Cytodex 3 (d) and P102-L (e). Figure f shows hMSC-TERT in a monolayer culture (tissue culture flask). All images were taken on the 4th day of cultivation. Because of the opacity of Biosilon, RapidCell and P102-L, the cells are just visible at the “horizon” of the microcarriers.

**Fig. (5):**
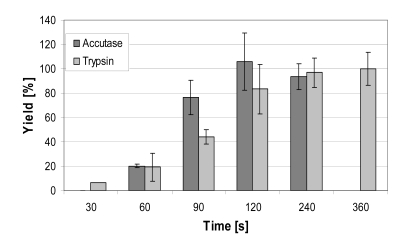
Cell yield of hMSC-TERT, cultured as monolayer in 6-well cell culture plates after treatment with trypsin and accutase for up to 6 minutes.

**Fig. (6):**
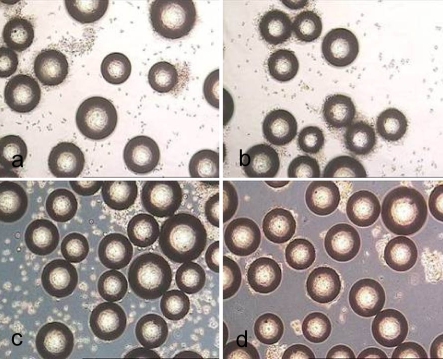
Biosilon after treatment for 10 minutes with trypsin (**a**), accutase (**b**) and accutase trypsin mix (**c**) as well as after treatment for 40 minutes with collagenase (**d**).

**Fig. (7):**
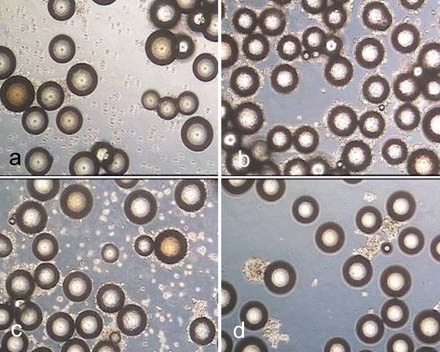
Rapidcell after treatment for 10 minutes with trypsin (**a**), accutase (**b**), accutase trypsin mix (**c**) and after treatment for 40 minutes with collagenase (**d**).

**Fig. (8):**
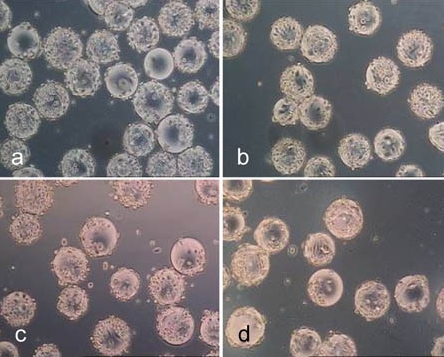
Cytodex 1 after treatment for 10 minutes with trypsin (**a**), accutase (**b**) and accutase trypsin mix (**c**) as well as after treatment for 40 minutes with collagenase (**d**).

**Fig. (9):**
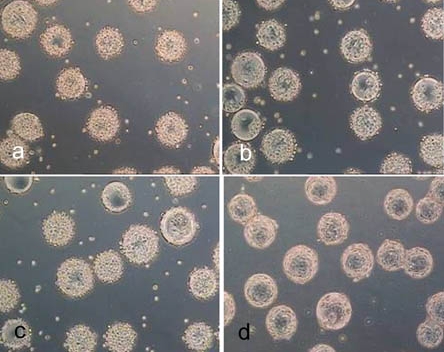
Cytodex 3 after treatment for 10 minutes with trypsin (**a**), accutase (**b**) and accutase trypsin mix (**c**) and after treatment for 40 minutes with collagenase (**d**).

**Fig. (10):**
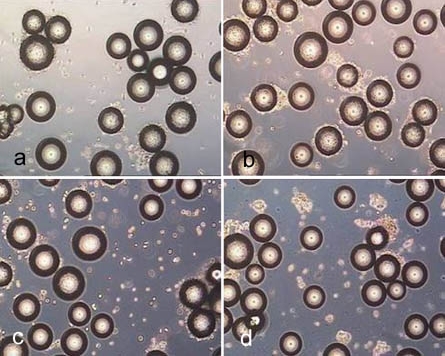
P102-L after treatment for 10 minutes with trypsin (**a**), accutase (**b**) and accutase trypsin mix (**c**) as well as after treatment for 40 minutes with collagenase (**d**).

**Table 1:** Properties and Materials of Spherical and Nonporous Microcarriers Used in this Work

Microcarrier	Manufacturer/Distributer	Specific surface [cm^2^/g]	Density [g/cm^3^]	Diameter [µm]	Material	Surface	Suface charge
**Biosilon**	Nunc	255	1.05	160-300	Polystyrene		-
**Cytodex 1**	Amersham Bioscience	4400	1.03	147-248	DEAE-Dextran	DEAE-Dextran	+
**Cytodex 3**	Amersham Bioscience	2700	10.4	141-210	DEAE-Dextran	Collagen	+
**P 102-L**	Hyclone	360	1.02	125-212	Polystyrene	Polystyrene	None
**Rapidcell**	MP Biomedicals	325	1.03	150-210		Glass	-
